# Association of fish intake and smoking with risk of rheumatoid arthritis and age of onset: a prospective cohort study

**DOI:** 10.1186/s12891-018-2381-3

**Published:** 2019-01-05

**Authors:** Jeffrey A. Sparks, Éilis J. O’Reilly, Medha Barbhaiya, Sara K. Tedeschi, Susan Malspeis, Bing Lu, Walter C. Willett, Karen H. Costenbader, Elizabeth W. Karlson

**Affiliations:** 1 Department of Medicine, Division of Rheumatology, Immunology and Allergy, Brigham and Women’s Hospital, 60 Fenwood Road, #6016U, Boston, MA 02115 USA; 2 Harvard Medical School, 25 Shattuck St, Boston, MA 02115 USA; 3 Department of Nutrition, Harvard T.H. Chan School of Public Health, 677 Huntington Ave, Boston, MA 02115 USA; 4 School of Public Health, University College Cork, University College, College Road, Cork, Ireland; 5 Hospital for Special Surgery, 535 East 70th Street, New York, NY 10021 USA; 6 Department of Medicine, Channing Division of Network Medicine, Brigham and Women’s Hospital, 181 Longwood Avenue, Boston, MA 02115 USA

**Keywords:** Rheumatoid arthritis, Fish, Diet, Inflammation, Omega-3 fatty acids, Smoking, Epidemiology

## Abstract

**Background:**

Prior studies suggest that fish may be protective for rheumatoid arthritis (RA) risk perhaps through the anti-inflammatory effect of omega-3 fatty acid, but this relationship has not been clearly established. Therefore, we investigated fish intake and RA risk by serologic status, age of onset, and smoking using a prospective cohort study with large sample size, repeated measures of dietary intake, and lengthy follow-up.

**Methods:**

We studied fish intake and RA risk among 166,013 women in two prospective cohorts, the Nurses’ Health Study (NHS, 1984–2014) and NHSII (1991–2015). Fish intake was assessed using food frequency questionnaires at baseline and every 4 years. Incident RA during follow-up and serologic status were determined by medical record review. Pooled Cox regression models estimated hazard ratios (HR) and 95% confidence intervals (CI) for RA (overall and by serologic status and age at diagnosis) for fish intake frequency. We tested for a smoking-fish interaction for RA risk.

**Results:**

During 3,863,909 person-years of follow-up, we identified 1080 incident RA cases. Increasing fish intake was not associated with all RA (≥4 servings/week: multivariable HR 0.93 [95%CI 0.67–1.28] vs. < 1 serving/month; *p* for trend = 0.42), seropositive RA (*p* for trend = 0.66), or seronegative RA (*p* for trend = 0.45), but had increased risk for RA diagnosed > 55 years old (*p* for trend = 0.037). Among women ≤55 years old, frequent fish intake (vs. infrequent) had HRs (95%CIs) of: 0.73 (0.52–1.02) for all RA, 0.85 (0.55–1.32) for seropositive RA, and 0.55 (0.32–0.94) for seronegative RA. Ever smokers with infrequent fish intake had highly elevated risk for RA onset ≤55 years (HR 2.59, 95%CI 1.65–4.06), while ever smokers with frequent fish intake had modestly elevated RA risk (HR 1.29, 95%CI 1.07–1.57; vs. never smokers/frequent fish intake; *p* for smoking-fish interaction = 0.039).

**Conclusion:**

In this large prospective cohort study, we found no clear protective effect of fish or marine omega-3 fatty acid intake on RA risk, overall or by serologic status. We found that fish intake attenuated the strong association of smoking for RA diagnosed ≤55 years of age, but this requires further study.

## Background

Progress continues to be made in identifying risk factors for rheumatoid arthritis (RA) [1]. Environmental factors may affect transitions from genetic risk to RA-related autoantibody development and between asymptomatic autoimmunity and clinical onset [2, 3]. While smoking is an important RA risk factor, many individuals who develop RA never smoked, so other factors likely contribute [4–8]. In particular, metabolic factors such as obesity and diet may be important for earlier RA onset [9–14].

Fish intake may decrease RA risk based on the anti-inflammatory properties of omega-3 polyunsaturated fatty acids (PUFAs), such as eicosapentaenoic acid (EPA), docosahexaenoic acid (DHA), and docosapentaenoic acid (DPA) [15]. Omega-3 PUFAs may have protective biologic effects in the phases prior to the onset of clinically-apparent RA [16–18]. After RA diagnosis, fish may have beneficial effects on disease activity and pain [19–22].

The association of fish intake with RA risk has been investigated previously, some studies showing inverse associations while others had no association [23–29]. A meta-analysis of fish intake and RA risk showed a reduction in RA risk by 20–24% (statistically non-significant) for 1–3 servings of fish/week compared to less [30]. These studies may not have been able to detect a modest association of dietary factors with RA, did not have data on confounders such as smoking, or did not investigate RA phenotypes based on serostatus or age at onset. No previous study has evaluated whether fish intake and RA risk may differ based on smoking status, the strongest environmental RA risk factor.

Therefore, we investigated fish intake and RA risk using the Nurses’ Health Study (NHS) and NHSII, two prospective cohort studies with lengthy follow-up and detailed dietary/covariate data. We hypothesized that increasing fish intake and marine omega-3 PUFAs would be associated with decreased RA risk, particularly earlier-onset RA or seropositive RA. We further aimed to investigate whether fish intake had a differential effect on RA by smoking status. We hypothesized that anti-inflammatory effects of fish intake may attenuate the elevated RA risk for smokers.

## Methods

### Study population

The NHS and NHSII are prospective cohorts of US women who were registered nurses at enrollment. The NHS enrolled 121,700 women aged 30–55 years in 1976; the NHSII enrolled 116,670 women aged 25–42 years in 1989. In both cohorts, women answered questionnaires at baseline and every 2 years to collect data on lifestyle, family history, diet, diagnoses, and medications.

In this study, the baseline for analysis in the NHS & NHSII were 1984 & 1991, respectively, when a comprehensive Food Frequency Questionnaire (FFQ) was first introduced. We excluded participants who reported a diagnosis of RA or other connective tissue disease (CTD) prior to the 1984 or 1991 questionnaires and those who did not answer the baseline FFQ. A total of 76,540 women in the NHS and 89,473 women in the NHSII were included. All aspects of the study the study comply with the Declaration of Helsinki and were approved by the Partners HealthCare Institutional Review Board.

### Dietary assessments

We used a semi-quantitative FFQ to measure food intake over the previous year [11, 31]. The FFQ has proven validity and reproducibility compared to food diaries and recall for a spectrum of dietary/nutritional factors [32, 33]. The FFQ ranks frequency of food/beverages on a scale ranging from never or < 1/month to ≥6 servings/day. Dietary intakes were assessed in the NHS in 1984, 1986, and every 4 years until 2010. In the NHSII, the FFQ was administered in 1991 and every 4 years until 2011. We used cumulative average intake to reflect long-term consumption and to reduce measurement error. For each questionnaire cycle evaluating RA risk, cumulative average intake was calculated by averaging the repeated dietary measure from baseline until that cycle. The cumulative average intake variable was time-varying such that all available dietary measures at each time point were taken into consideration to predict RA risk in the subsequent questionnaire cycle. For example, only the fish intake measured at baseline in 1984 was considered for RA risk in the window of 1984 to 1986 in the NHS since no previous measures were available at that time point. However, a total of 8 dietary measures were used for cumulative average variable on the 2012 questionnaire cycle assessing for RA risk between 2012 and 2014. We did not analyze simple updated time-varying exposures since short-term dietary changes are less likely to impact chronic disease risk than long-term dietary habits. For categorical variables, the cumulative average intake was calculated as a continuous measure prior to categorization.

Our primary exposure was fish intake frequency. The FFQ included four items related to fish intake: 1) dark meat fish (mackerel, salmon, sardines, bluefish, or swordfish, 3–5 oz; 84–140 g); 2) canned tuna (3–4 oz; 84–112 g); 3) other fish (3–5 oz; 84–140 g); and 4) shrimp, lobster, or scallops as main dish. One fish item was added in the 1994 & 1998 FFQs: breaded fish cakes, pieces, or fish sticks (1 serving). We calculated the servings of total fish consumed at each questionnaire cycle. As the primary exposure, we considered five categories for total fish intake frequency: none to < 1/month, 1/month to < 1/week, 1 to < 2.5/week, 2.5 to < 4/week, and ≥ 4/week. We chose these categories based on recommendations of fish intake and their use in previous reports investigating the association of fish intake with chronic disease risk [34, 35]. To investigate a threshold effect of fish intake on RA risk, we dichotomized this variable into infrequent (none to < 1/month) and frequent fish intake (≥1/month).

To investigate the effect of nutrients on RA risk, we analyzed the effect of marine omega-3 PUFAs intake. We considered marine omega-3 PUFAs as the sum of EPA, DHA, and DPA from diet and supplements. We did not consider these PUFAs separately since high correlation limited assessment of their independent effects. We calculated the total marine omega-3 PUFAs in each cycle as previously described [36]. Since there are no clear cutpoints for omega-3 PUFAs and RA risk, we analyzed quartiles of marine omega-3 PUFA intake.

### Identification of incident RA

Women who self-reported RA or other CTD were administered a screening questionnaire [37]. For those who screened positive, medical records were obtained to verify the diagnosis and collect symptom/diagnosis dates and serostatus. All records were reviewed by two rheumatologists and all cases had RA by accepted criteria [38, 39]. We defined seropositive RA as positive rheumatoid factor (RF) or anti-cyclic citrullinated peptide (anti-CCP). We relied on clinical testing so were unable to perform analyses based solely on anti-CCP serostatus since this test was not used clinically when most of the follow-up occurred. The end of follow-up was June 1, 2014 for the NHS and June 1, 2015 for the NHSII.

### Covariates

#### Sociodemographic, lifestyle, and reproductive factors

Data on time-varying covariates were obtained through biennial questionnaires. We considered variables that may be associated with fish intake and RA based on prior literature [4, 9, 10, 40, 41]. Among past/current smokers, we categorized smoking intensity as 1–14 cigarettes/day or ≥ 15 cigarettes/day. We calculated smoking pack-years and dichotomized as never/≤10 pack-years or > 10 pack-years [8]. Body mass index (BMI) was categorized as underweight/normal (< 25.0 kg/m^2^), overweight (25 to < 30 kg/m^2^), or obese (≥30 kg/m^2^). Physical activity was measured using a validated survey and converted into weekly metabolic equivalents [42]. We categorized parity and breastfeeding duration into a single variable: nulliparous, parous/breastfeeding for none to < 1 month, parous/breastfeeding for 1 to < 12 months, or parous/breastfeeding ≥12 months. Menopausal status and postmenopausal hormone (PMH) use was categorized as: premenopausal, postmenopausal/never PMH use, or postmenopausal/ever PMH use. We considered household income derived from the US Census tract median income at the level of zip code, categorized into quartiles.

#### Other dietary factors

Energy intake was obtained by summing the daily nutritional content of all FFQ items as a continuous variable (kilocalories/day). Alcohol intake was categorized as: none to < 5 g/day, 5 to < 10 g/day, or ≥ 10 g/day. In analyses that included marine omega-3 PUFAs, we also included other PUFA types (alpha-linoleic acid, omega-6 PUFAs), fatty acids (*trans*, saturated, and monounsaturated), and macronutrients (protein and carbohydrates) as continuous variables (g/day).

### Statistical analysis

We described participants in each cohort according to the baseline distribution of five categories of fish intake using mean and standard deviation (SD) for continuous variables and proportions for categorical variables. We pooled both cohorts into a single analysis to improve statistical efficiency given planned secondary and interaction analyses with low numbers of subgroup outcomes.

Since we previously found differences in metabolic/dietary RA risk factors related to age ≤ 55 or > 55 years old (chosen related to clinical observations of differing RA phenotypes related to age at diagnosis and as an approximation of menopause), we compared clinical characteristics of RA cases by these ages at diagnosis [9, 11, 13, 14]. Median time from first symptoms to diagnosis were compared by the Wilcoxon-rank sum test. We compared the distribution of 1987 ACR criteria using the chi-square test.

The primary analysis assessed the association of 5 categories of fish intake with all RA risk as well as by serostatus and stratified by age at diagnosis (≤55 or > 55 years). Other secondary analyses investigated the association of fish as a binary variable (frequent [≥1 serving/month] or infrequent [none to < 1 serving/month] intake) with RA risk overall and by serostatus and age stratification. We used Cox proportional hazards models to obtain hazard ratios (HR) and 95% confidence intervals (CI) for the association of fish intake with RA risk, with the lowest category of fish intake as the reference group. Person-years commenced from the return date of the baseline questionnaire to the end of follow-up, death, or censor, whichever came first. Women were censored for self-reported CTD not confirmed to be RA. We considered the lowest category of fish intake as the reference group. In base models, we adjusted for age, questionnaire period, cohort, and energy. We built multivariable models based on the primary analysis and included covariates that were associated with both fish intake and RA risk. The final multivariable model included the base factors as well as median household income, smoking, BMI, and alcohol intake. We calculated *p* for trend using the median value within each category as a continuous variable in the model.

We investigated the association of quartiles of marine omega-3 PUFAs with the RA outcomes. We considered women in the lowest quartile of intake as the reference group. In these regression models, we also adjusted for other types of PUFAs, fatty acids, and macronutrients to determine the independent of effect of marine omega-3 fatty acids. Since total energy was included, we did not additionally include carbohydrates since all other macronutrients were present.

LLastly, we investigated an interaction between smoking and fish intake, both considered as binary variables (never/ever smoking and frequent/infrequent fish intake). We investigated all RA, as well as stratified analyses based on age at diagnosis (≤55 or > 55 years). We created a cross-classified variable as our main exposure, with never smoking/frequent fish intake as the reference group. We obtained *p* for multiplicative interactions from an interaction term in the model.

We verified the proportional hazards assumption in all analyses by comparing nested models with and without interaction terms of follow-up time and exposure status. A two-sided *p* < 0.05 was considered statistically significant.

## Results

Baseline characteristics of both cohorts according to 5 categories of fish intake are shown in Table 1. In both cohorts (*n* = 166,013), fish intake of none to < 1 serving/month occurred infrequently (5.7% in NHS and 9.3% in NHSII). Age, race, US region, and reproductive factors were similar regardless of fish intake. Daily energy intake increased as fish intake increased. Those who ate fish frequently were more likely to be ever smokers and had higher income, alcohol intake, and BMI compared to those who infrequently ate fish.

**Table 1:** Baseline age-standardized characteristics of participants in the Nurses’ Health Study in 1984 and Nurses’ Health Study II in 1991 categorized by total fish intake (*n* = 166,013)

Characteristics	Total fish servings									
	NHS (*n* = 76,540)					NHSII (*n* = 89,473)				
	None to < 1/month	1/month to < 1/week	1 to < 2.5/week	2.5 to < 4/week	≥4/week	None to < 1/month	1/month to < 1/week	1 to < 2.5/week	2.5 to < 4/week	≥4/week
Participants, n (%)	4343 (5.7)	10,165 (13.3)	40,913 (53.5)	7284 (9.5)	13,835 (18.1)	8296 (9.3)	15,470 (17.3)	46,496 (52.0)	6933 (7.7)	12,278 (13.7)
Age in years, mean (SD)^a^	50.8 (7.3)	50.1 (7.3)	50.1 (7.2)	51.1 (7.0)	50.8 (7.0)	35.6 (4.8)	35.9 (4.7)	36.1 (4.6)	36.4 (4.5)	36.4 (4.6)
Calorie intake/day (SD)	1554 (526)	1605 (514)	1737 (514)	1835 (537)	1868 (543)	1593 (532)	1640 (521)	1791 (527)	1914 (544)	2025 (556)
White race, %	97.3	97.3	97.6	96.0	96.8	93.1	93.4	93.5	91.2	90.1
Median household income quartile, %^b^										
Q1 – lowest income	30.7	28.2	24.5	22.1	21.1	25.9	25.6	24.4	21.4	22.8
Q2	26.9	25.6	25.7	23.0	23.2	26.7	26.2	25.1	22.4	23.0
Q3	22.3	24.4	25.0	25.3	27.3	25.3	25.6	25.3	24.6	25.2
Q4 – highest income	20.1	21.7	24.8	29.6	28.4	22.2	22.7	25.2	31.7	29.0
Smoked > 10 pack years, %	36.6	37.0	36.2	36.0	36.8	16.1	15.5	15.8	16.2	16.8
Ever smoked, %	53.2	54.4	55.8	57.8	59.5	32.8	32.7	34.4	37.8	36.8
Smoking status/intensity, %										
Never	46.8	45.6	44.2	42.2	40.5	67.2	67.3	65.6	62.2	63.2
Past, 1–14 cigarettes/day	12.3	13.6	15.8	17.1	18.6	10.7	11.1	12.6	14.7	13.9
Past, ≥15 cigarettes/day	13.5	14.3	15.4	17.4	20.0	9.6	9.3	9.7	10.8	11.1
Current, 1–14 cigarettes/day	7.2	7.4	7.3	8.1	7.9	5.0	5.1	5.5	6.0	5.8
Current, ≥15 cigarettes/day	20.2	19.1	17.2	15.2	13.0	7.5	7.2	6.5	6.3	6.0
Parity/breastfeeding duration, %										
Nulliparous	7.0	6.0	5.4	6.6	6.0	30.5	25.5	24.5	29.4	30.8
Parous/none to < 1 month	45.2	43.6	42.7	40.3	41.6	14.8	14.8	13.2	11.5	11.6
Parous/1 to < 12 months	28.7	31.0	30.8	31.3	31.1	22.6	24.7	25.0	23.5	23.0
Parous/≥12 months	14.7	14.8	16.9	17.1	16.6	24.0	27.3	29.9	28.0	26.5
Menopausal status/postmenopausal hormone use										
Premenopausal	39.0	40.1	40.8	40.1	39.5	91.6	91.9	92.6	92.8	92.3
Postmenopausal/never use	31.7	31.4	31.5	31.6	31.8	3.8	3.8	3.4	3.3	3.4
Postmenopausal/ever use	26.4	25.8	25.4	26.0	26.5	4.3	4.1	3.8	3.6	4.1
Body mass index category, %										
Underweight/normal (< 25 kg/m^2^)	64.1	63.3	61.8	60.7	54.1	68.2	68.1	67.6	67.7	63.3
Overweight (25 to < 30 kg/m^2^)	23.6	24.5	25.7	26.2	30.5	18.7	18.7	19.9	20.0	22.2
Obese (≥30 kg/m^2^)	12.3	12.2	12.5	13.1	15.3	13.1	13.1	12.5	12.2	14.5
Alcohol intake, %										
None to < 5 g/day	73.9	70.2	63.8	58.5	63.0	84.5	82.8	79.6	74.7	77.5
5 to < 10 g/day	8.6	9.2	12.1	12.6	12.5	8.5	9.3	10.9	14.0	11.3
≥ 10 g/day	17.5	20.5	24.1	28.9	24.5	7.0	7.9	9.5	11.3	11.2

Missing values are not shown

^a^ Value is not age-standardized

^b^ Median household income was obtained from US Census-tract data in 1986 for the NHS

During a total of 3,863,909 person-years of follow-up, there were 1080 incident RA cases. Of these, 672 (62.2%) were seropositive and 408 (37.8%) were seronegative. Table 2 shows RA clinical characteristics at presentation according to age at RA diagnosis. Women with RA diagnosed at age ≤ 55 years tended to have more 1987 ACR criteria (*p* = 0.016) and were more likely to be seropositive (*p* = 0.020) than women diagnosed with RA at age > 55 years.

**Table 2:** Characteristics of 1080 incident rheumatoid arthritis cases at presentation based on diagnosis before or after 55 years of age

	RA diagnosed ≤ 55 years (*n* = 491)	RA diagnosed > 55 years (*n* = 589)	*p* value
Median time from first symptoms to diagnosis, months (IQR)	7 (3, 13)	6 (2, 13)	0.17
Number of 1987 ACR criteria, %			0.016
4	45.2	53.5	
5	43.0	34.8	
6–7	11.8	11.7	
Seropositive^a^, %	66.0	59.1	0.020
Radiographic changes, %	24.6	28.7	0.13
Hand arthritis, %	98.8	98.0	0.30
Symmetric arthritis, %	96.7	96.9	0.85
≥3 joint areas affected, %	95.8	93.5	0.090
> 1 h of morning stiffness, %	75.8	73.9	0.47
Rheumatoid nodules, %	10.0	8.8	0.52

^a^ Seropositive was defined as positive rheumatoid factor and/or anti-cyclic citrullinated peptide on medical record review

ACR, American College of Rheumatology; IQR, interquartile range; RA, rheumatoid arthritis

The association between 5 categories of cumulative average fish intake and RA risk is shown in Table 3. Compared to infrequent fish intake (none to < 1 serving/month), there was no association with more frequent fish intake categories and all RA risk. In the base model adjusted for age, questionnaire period, energy, and cohort, women with fish intake ≥4 servings/week had HR for all RA of 0.92 (95%CI 0.66–1.27; *p* for trend = 0.48). In the multivariable model additionally adjusted for household income, smoking, BMI, and alcohol intake, the HR for all RA was 0.93 (95%CI 0.67–1.28; *p* for trend = 0.42). We found no association of fish intake with seropositive RA (*p* for trend = 0.66), seronegative RA (*p* for trend = 0.45), and all RA among women aged ≤55 years (*p* for trend = 0.29). While there was no statistically significant trend, the point estimate of the HR tended to be < 1 compared to none to infrequent fish intake for all categories in all RA, seropositive RA, seronegative RA, and all RA diagnosed ≤55 years old. However, increasing fish intake across 5 categories was associated with increased RA risk among women aged > 55 years (*p* for trend = 0.037). In subgroup analyses, there was no association across categories of fish intake with risk of seropositive RA among women ≤55 years (*p* for trend = 0.33) or seronegative RA ≤55 years (*p* for trend = 0.99).

**Table 3:** Hazard ratios for rheumatoid arthritis phenotypes according to categories of cumulative average total fish intake in the Nurses’ Health Study and Nurses’ Health Study II (*n* = 166,013)

	Total fish servings					
	None to < 1/month	1/month to < 1/week	1 to < 2.5/week	2.5 to < 4/week	≥4/week	*p* for trend
	HR (95%CI)	HR (95%CI)	HR (95%CI)	HR (95%CI)	HR (95%CI)	
All RA						
Cases/person-years	53/185,861	175/705,447	513/1,888,251	200/649,928	139/434,424	
Age-adjusted model^a^	1.00 (Ref)	0.82 (0.60–1.11)	0.83 (0.62–1.11)	0.87 (0.64–1.19)	0.92 (0.66–1.27)	0.48
Multivariable model^b^	1.00 (Ref)	0.82 (0.60–1.12)	0.84 (0.63–1.12)	0.89 (0.65–1.22)	0.93 (0.67–1.28)	0.42
Seropositive RA						
Cases/person-years	34/185,640	116/704,320	308/1,884,304	133/648,527	81/433,284	
Age-adjusted model^a^	1.00 (Ref)	0.86 (0.59–1.27)	0.80 (0.56–1.15)	0.95 (0.64–1.39)	0.87 (0.57–1.31)	0.72
Multivariable model^b^	1.00 (Ref)	0.86 (0.59–1.27)	0.82 (0.57–1.17)	0.97 (0.66–1.43)	0.88 (0.58–1.33)	0.66
Seronegative RA						
Cases/person-years	19/185,340	59/703,876	205/1,883,542	67/648,136	58/432,966	
Age-adjusted model^a^	1.00 (Ref)	0.74 (0.44–1.25)	0.88 (0.54–1.41)	0.76 (0.45–1.27)	0.99 (0.59–1.69)	0.48
Multivariable model^b^	1.00 (Ref)	0.74 (0.44–1.25)	0.88 (0.55–1.42)	0.77 (0.46–1.29)	1.01 (0.59–1.71)	0.45
All RA among ≤ 55 years						
Cases/person-years	37/134,613	113/458,908	213/1,072,596	74/301,548	54/224,372	
Age-adjusted model^a^	1.00 (Ref)	0.85 (0.59–1.24)	0.64 (0.45–0.91)	0.72 (0.48–1.08)	0.71 (0.46–1.10)	0.25
Multivariable model^b^	1.00 (Ref)	0.86 (0.59–1.25)	0.66 (0.46–0.94)	0.75 (0.50–1.12)	0.72 (0.47–1.11)	0.29
All RA among > 55 years						
Cases/person-years	16/52,659	62/252,365	300/828,551	126/352,538	85/211,857	
Age-adjusted model^a^	1.00 (Ref)	0.82 (0.47–1.42)	1.19 (0.72–1.98)	1.19 (0.70–2.01)	1.30 (0.75–2.23)	0.043
Multivariable model^b^	1.00 (Ref)	0.82 (0.47–1.42)	1.21 (0.73–2.00)	1.21 (0.71–2.05)	1.32 (0.76–2.27)	0.037

There were 1080 cases in 3,863,909 total person-years for all RA analyses. There were 672 cases in 3,856,074 total person-years for seropositive RA analyses

There were 408 cases in 3,853,860 total person-years for seronegative RA analyses. There were 491 cases in 2,192,037 total person-years for all RA among ≤55 years analyses. There were 589 cases in 1,697,970 total person-years for all RA among > 55 years analyses

The exposure period was 1984 to 2012 for cases occurring until 2014 in the NHS; the exposure period was 1991 to 2013 for cases occurring until 2015 in the NHSII

^a^ Adjusted for age, questionnaire period, cohort, and total energy intake (continuous)

^b^ Multivariable models were adjusted for age, questionnaire period, cohort, total energy intake (continuous), median household income (quartiles), cigarette smoking (never, past 1–14/day, past ≥15/day, current 1–14/day, current ≥15/day), body mass index category (underweight/normal, overweight, obese), and alcohol intake (never to < 5, 5 to < 10, ≥10 g/day)

*CI* confidence interval, *HR* hazard ration, *RA* rheumatoid arthritis

Table 4 shows the results evaluating a threshold effect for RA risk where infrequent fish intake was considered as none to < 1 serving/month and frequent fish intake was ≥1 serving/month. Compared to infrequent fish intake, frequent intake was not associated with all RA (multivariable HR 0.85, 95%CI 0.64–1.13), seropositive RA (HR 0.86, 95%CI 0.60–1.22), or seronegative RA (HR 0.85, 95%CI 0.53–1.35). Among women aged ≤55 years, frequent fish intake was significantly associated with decreased all RA risk in the base model (HR 0.71, 95%CI 0.50–1.00, *p* = 0.048). In the multivariable model, the association of frequent fish intake with reduced RA risk was similar but not statistically significant (HR 0.73, 95%CI 0.52–1.02, *p* = 0.066). In one subgroup, there was a statistically significant protective effect of frequent fish intake for seronegative RA diagnosed ≤55 years of age (HR 0.55, 95%CI 0.32–0.94). Frequent fish intake was not associated with RA diagnosed > 55 years (HR 1.14, 95%CI 0.69–1.88).

**Table 4:** Hazard ratios for rheumatoid arthritis phenotypes according to dichotomized cumulative average total fish intake in the Nurses’ Health Study and Nurses’ Health Study II (*n* = 166,013)

	Total fish servings		
	None to < 1/month (low)	≥1/month (high)	*p* value
	HR (95%CI)	HR (95%CI)	
All RA			
Cases/person-years	53/185,861	1027/3,678,049	
Age-adjusted model^a^	1.00 (Ref)	0.84 (0.64–1.11)	0.23
Multivariable model^b^	1.00 (Ref)	0.85 (0.64–1.13)	0.27
Seropositive RA			
Cases/person-years	34/185,640	638/3,670,434	
Age-adjusted model^a^	1.00 (Ref)	0.84 (0.59–1.20)	0.34
Multivariable model^b^	1.00 (Ref)	0.86 (0.60–1.22)	0.39
Seronegative RA			
Cases/person-years	19/185,340	389/3,668,519	
Age-adjusted model^a^	1.00 (Ref)	0.84 (0.53–1.34)	0.47
Multivariable model^b^	1.00 (Ref)	0.85 (0.53–1.35)	0.48
All RA among ≤ 55 years			
Cases/person-years	37/134,613	454/2,057,424	
Age-adjusted model^a^	1.00 (Ref)	0.71 (0.50–1.00)	0.048
Multivariable model^b^	1.00 (Ref)	0.73 (0.52–1.02)	0.066
Seropositive RA among ≤ 55 years			
Cases/person-years	22/134,452	302/2,053,251	
Age-adjusted model^a^	1.00 (Ref)	0.83 (0.53–1.29)	0.40
Multivariable model^b^	1.00 (Ref)	0.85 (0.55–1.32)	0.47
Seronegative RA among ≤ 55 years			
Cases/person-years	15/134,186	152/2,051,358	
Age-adjusted model^a^	1.00 (Ref)	0.54 (0.31–0.92)	0.024
Multivariable model^b^	1.00 (Ref)	0.55 (0.32–0.94)	0.029
All RA among > 55 years			
Cases/person-years	16/52,659	573/1,645,311	
Age-adjusted model^a^	1.00 (Ref)	1.13 (0.69–1.87)	0.62
Multivariable model^b^	1.00 (Ref)	1.14 (0.69–1.88)	0.60

^a^Adjusted for age, questionnaire period, cohort, and total energy intake (continuous)

^b^Multivariable models were adjusted for age, questionnaire period, cohort, total energy intake (continuous), median household income (quartiles), cigarette smoking (never, past 1–14/day, past ≥15/day, current 1–14/day, current ≥15/day), body mass index category (underweight/normal, overweight, obese), and alcohol intake (never to < 5, 5 to < 10, ≥10 g/day)

*CI* confidence interval, *HR* hazard ratio, *RA* rheumatoid arthritis

Table 5 shows the results investigating quartiles of marine omega-3 PUFA (EPA, DHA, and DPA) intake with RA risk. There was no association of quartiles of marine omega-3 PUFAs with all RA (*p* for trend = 0.28), seropositive RA (*p* for trend = 0.19), seronegative RA (*p* trend = 0.92), or among those aged ≤55 years (*p* for trend = 0.40). There was no association with RA among those aged > 55 years in the base model (*p* for trend = 0.097), but the multivariable model was statistically significant (*p* for trend = 0.031). There was no association from lowest to highest quartile of marine omega-3 PUFA intake with risk of seropositive RA among women ≤55 years (*p* for trend = 0.19) or seronegative RA among women ≤55 years (*p* for trend = 0.19) in these subgroup analyses.

**Table 5:** Hazard ratios for rheumatoid arthritis phenotypes according to quartiles of marine omega-3 polyunsaturated fatty acid^a^ intake in the Nurses’ Health Study and Nurses’ Health Study II (*n* = 166,013)

	Marine omega-3 polyunsaturated fatty acid intake^a^				
	Quartile 1	Quartile 2	Quartile 3	Quartile 4	*p* for trend
	HR (95%CI)	HR (95%CI)	HR (95%CI)	HR (95%CI)	
All RA					
Cases/person-years	251/945,683	269/969,619	279/980,365	281/968,243	
Age-adjusted model^b^	1.00 (Ref)	1.03 (0.87–1.22)	1.05 (0.88–1.24)	1.04 (0.88–1.24)	0.65
Multivariable model^c^	1.00 (Ref)	1.05 (0.88–1.25)	1.09 (0.91–1.31)	1.12 (0.91–1.37)	0.28
Seropositive RA					
Cases/person-years	167/943,993	153/967,552	177/978,198	175/966,331	
Age-adjusted model^b^	1.00 (Ref)	0.88 (0.70–1.09)	1.00 (0.81–1.23)	0.97 (0.79–1.21)	0.82
Multivariable model^c^	1.00 (Ref)	0.92 (0.73–1.15)	1.08 (0.86–1.36)	1.12 (0.87–1.43)	0.19
Seronegative RA					
Cases/person-years	84/943,360	116/967,367	102/977,670	106/965,464	
Age-adjusted model^b^	1.00 (Ref)	1.34 (1.01–1.77)	1.14 (0.86–1.53)	1.18 (0.89–1.58)	0.64
Multivariable model^c^	1.00 (Ref)	1.31 (0.98–1.75)	1.12 (0.82–1.51)	1.14 (0.81–1.59)	0.92
All RA among ≤ 55 years					
Cases/person-years	125/565,147	139/564,890	120/548,635	107/513,366	
Age-adjusted model^b^	1.00 (Ref)	1.10 (0.87–1.41)	0.96 (0.75–1.24)	0.90 (0.69–1.17)	0.23
Multivariable model^c^	1.00 (Ref)	1.10 (0.86–1.41)	0.98 (0.75–1.27)	0.93 (0.69–1.25)	0.40
All RA among > 55 years					
Cases/person-years	126/386,863	130/411,627	159/438,231	174/461,250	
Age-adjusted model^b^	1.00 (Ref)	0.96 (0.75–1.23)	1.12 (0.89–1.42)	1.16 (0.92–1.47)	0.097
Multivariable model^c^	1.00 (Ref)	1.00 (0.77–1.28)	1.20 (0.93–1.54)	1.29 (0.98–1.69)	0.031

^a^Dietary and supplementary intake of eicosapentaenoic acid (EPA), docosahexaenoic acid (DHA), and docosapentaenoic acid (DPA) were considered as marine omega-3 polyunsaturated fatty acids

^b^Adjusted for age, questionnaire period, cohort, and total energy intake (continuous)

^c^Multivariable models were adjusted for age, questionnaire period, cohort, total energy intake (continuous), median household income (quartiles), cigarette smoking (never, past 1–14/day, past ≥14/day, current 1–14/day, current ≥14/day), body mass index category (underweight/normal, overweight, obese), alcohol intake (never to < 5, 5 to < 10, ≥10 g/day), alpha-linolenic acid (g/d), *trans* fatty acids (g/d), saturated fatty acids (g/d), monounsaturated fatty acids (g/d), omega-6 polyunsaturated fatty acids (g/d), and protein (g/d)

*CI* confidence interval, *HR* hazard ratio, *RA* rheumatoid arthritis

**Fig. 1:**
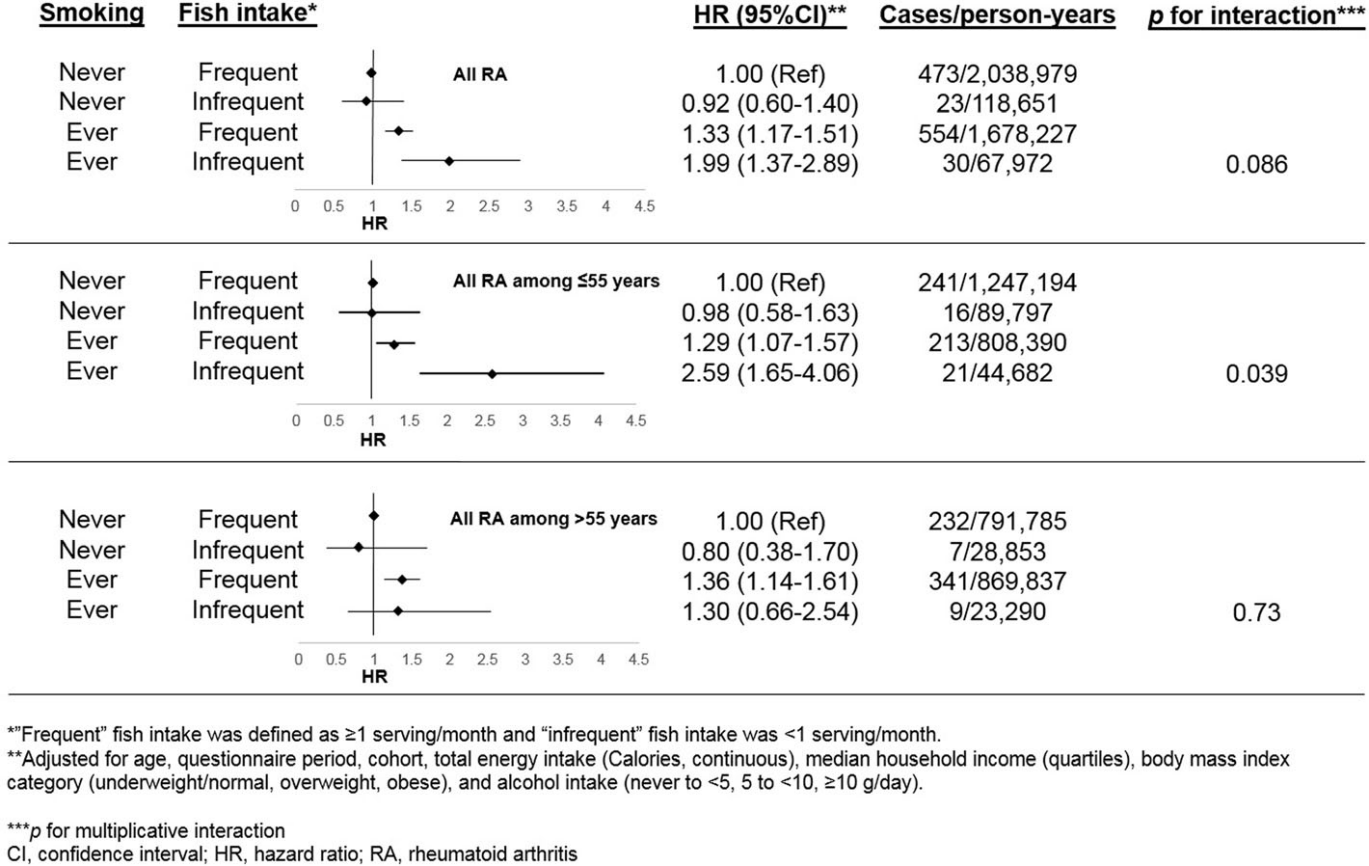
Interaction between smoking status and fish intake on risk of rheumatoid arthritis and stratified by age

Fig. 1 shows the results comparing RA risk based on smoking status and fish consumption. Ever smokers with infrequent fish intake had increased all RA risk (HR 1.99, 95%CI 1.37–2.89) compared to never smokers with frequent fish intake. However, there was no statistical interaction between fish and smoking for all RA (*p* for interaction = 0.086). There was a statistically significant interaction between fish and smoking for RA risk among women aged ≤55 years. In this subgroup, ever smokers with infrequent fish intake had very elevated RA risk (HR 2.59, 95%CI 1.65–4.06) while ever smokers with frequent fish intake had modestly increased RA risk (HR 1.29, 95%CI 1.07–1.57; vs. never smokers with frequent fish intake; *p* for interaction = 0.039). There was no smoking-fish interaction among those aged > 55 years (*p* for interaction = 0.73).

## Discussion

In this prospective study investigating fish intake and RA risk among 166,013 women with over 3.8 million person-years of follow-up, we found that increasing frequency of fish intake was not associated with overall RA risk or serologic RA phenotypes. Unlike previous studies, we found no evidence for a protective association between marine omega-3 PUFA intake and RA. In one subgroup, we did find a modest inverse association of fish intake above a threshold above 1 serving/month with RA risk, but only for earlier onset seronegative RA (age ≤ 55 years). While our results suggest that fish intake may attenuate the increased RA risk among ever smokers in this younger age group, fish intake may actually increase RA risk among older individuals > 55 years of age. Overall, these results do not support a strong effect of fish or omega-3 fatty acid intake on RA risk.

Our study adds to the literature of a relationship between fish intake and RA risk [30]. Several case-control studies suggested that fish intake, particularly dark meat/oily fish, was associated with decreased RA risk [23–25, 28]. These studies may have been limited due to recall bias and were conducted in coastal geographic areas with high fish intake (US Pacific Northwest, Greece, and Sweden) [23–25, 28]. Only one of these case-control studies adjusted for smoking [28]. Two studies investigated fish and seropositive RA risk but were underpowered for definitive conclusions [24, 28].

Fish intake and RA risk was investigated previously in cohort studies [26, 27, 29]. In a Danish study, fish intake on a single FFQ was not associated with RA, but there were few RA outcomes, so it may have been underpowered [26]. The Swedish Mammography cohort that analyzed two FFQ assessments may have suggested a modest, albeit statistically non-significant, decreased RA risk for fish intake ≥1 serving/week (RR 0.71, 95%CI 0.48–1.04), but analyses were not stratified by age [29]. They found that omega-3 PUFA intake above the threshold of the first quartile (> 0.21 g/day) was protective for RA risk (RR 0.65, 95%CI 0.48–0.90) [29]. Our omega-3 PUFA intake analyses did not detect a protective effect. While that study adjusted for smoking, the authors did not stratify by age at diagnosis, and fish-smoking interactions were not studied [29]. Our group previously investigated fish intake, among other dietary factors, and RA risk in the NHS and found no association [27]. This present report nearly doubles the number of incident RA cases by extending follow-up by 12 years and including the NHSII, allowing for increased power as well as subgroup analyses of RA phenotypes and smoking-fish interaction analyses. Despite adequate sample size and number of outcomes, our study did not find a protective effect on seropositive RA.

Several investigations in the Studies of the Etiology of RA (SERA) suggest that omega-3 PUFAs may be particularly important in the preclinical transitions preceding clinical RA presentation. Among those with the shared epitope or RA-related autoantibodies, omega-3 supplement use and erythrocyte-bound PUFAs were inversely associated with anti-CCP, RF positivity, and inflammatory arthritis [16–18]. Another SERA study reported that early age and smoking are both important factors for inflammatory arthritis [43]. Our results suggest that fish intake may be relatively more beneficial among younger/middle-aged women who were ever smokers. Since smokers may have increased systemic inflammation, long-term fish intake may exert effects by lowering inflammation once present due to smoking or other factors. A European nested case-control study showed that erythrocyte-bound omega-6 PUFAs, but not omega-3 PUFAs, were inversely associated with RA risk, so the effect of PUFAs on RA is still unclear [44]. Given the number of comparisons pursued in our study, the finding of the smoking-fish interaction should be considered as exploratory. While erythrocyte-bound assays and intake on FFQ have both been validated as measures of omega-3 PUFAs, they are only modestly correlated with each other, and it is unclear how much fish intake is needed for a meaningful increase in erythrocyte measures of omega-3 PUFAs [32, 45]. Therefore, the differences in our findings and the studies in SERA may be related to these different measures of omega-3 PUFAs in addition to studying different outcomes (surrogates of RA such as presence of RA-related autoantibodies in SERA vs. onset of RA in our study).

Strengths of our study include a large sample size, long follow-up, and many RA outcomes with detailed dietary/covariate data. We were able to investigate fish intake and risk of RA phenotypes based on serostatus and age at diagnosis. We used a prospective cohort design, so time-varying fish intake measures were collected prior to RA onset and less likely to be affected by recall bias. Women had multiple repeated measures of diet during follow-up and we analyzed the cumulative average fish intake to reflect long-term dietary intake. A recent study showed that unaffected relatives of RA were patients unaware that fish intake may affect RA risk and were motivated to change behaviors after a personalized RA risk educational intervention [46, 47]. However, this dietary recommendation may have at best only a modest effect on RA risk.

Our study has limitations to consider. The cohorts included mostly white and educated women who were healthy and working at baseline, so may not be generalizable. However, about two-thirds of patients with RA are women but it is unclear whether fish intake would have a similar effect on RA risk for men. While the FFQ has been widely used as a survey instrument to collect dietary data, it may not be completely accurate. Moreover, given the period of observation, fish consumption was low and often in the form of tuna, rather than other types of fresh fish, which likely has more powerful effects on increasing omega-3 PUFA levels. We were able to adjust for important covariates, such as smoking and BMI, but residual confounding is still possible. While we found a modest inverse association between fish intake and RA risk among women ≤55 years of age, we found no association of omega-3 PUFA intake with RA, even among this subgroup. It is possible that these could be chance findings related to multiple comparisons, though we pre-specified hypotheses based on literature [11, 43]. Our group has found differences related to elevated BMI and other dietary factors for RA occurring at less than 55 years which may be related to biologic differences based on menopause [9, 11, 13, 14]. Most of the participants in the SERA studies investigating omega-3 PUFAs and RA outcomes were younger than 55 years, but these studies were not specifically restricted to premenopausal women [16–18]. Less than 10% of women in both cohorts were categorized as having infrequent fish intake, so most women that were analyzed met this threshold and there were only a few cases that occurred in the infrequent fish intake category. However, in the main analysis investigating 5 categories of fish intake, there was no clear effect of higher categories of fish intake for reducing RA risk. We found no protective association of fish intake with seropositive RA and actually found an increased risk for RA diagnosed at > 55 years, both contrary to our hypothesis and previous studies [16, 24].

## Conclusions

In conclusion, our study did not find an association of fish intake or marine omega-3 PUFA intake with overall, seropositive, or seronegative RA risk. We found a suggestion that increasing fish intake may actually increase RA risk for women diagnosed at age > 55 years. We identified a smoking-fish interaction, such that ever smokers with frequent fish intake had only a modestly increased earlier-onset RA risk compared to the very elevated RA risk of smokers with infrequent fish intake. Our study does not provide evidence to recommend fish or omega-3 fatty acid intake to those at risk for RA.

## Abbreviations

Anti-CCP: Anti-cyclic citrullinated peptide
BMI: Body mass index
CI: Confidence interval
CSQ: Connective tissue disease screening questionnaire
CTD: Connective tissue disease
DHA: Docosahexaenoic acid
DPA: Docosapentaenoic acid
EPA: Eicosapentaenoic acid
FFQ: Food frequency questionnaire
HR: Hazard ratio
NHS: Nurses’ health study
NHSII: Nurses’ health study II
PMH: Postmenopausal hormone
PUFA: Polyunsaturated fatty acid
RA: Rheumatoid arthritis
RF: Rheumatoid factor
SD: Standard deviation
